# Engineering microorganisms based on molecular evolutionary analysis: a succinate production case study

**DOI:** 10.1111/eva.12186

**Published:** 2014-09-02

**Authors:** Xianghui Ma, Xinbo Zhang, Baiyun Wang, Yufeng Mao, Zhiwen Wang, Tao Chen, Xueming Zhao

**Affiliations:** 1 Department of Biochemical Engineering, School of Chemical Engineering and Technology, Tianjin UniversityTianjin, China; 2 Key Laboratory of Systems Bioengineering, Ministry of Education, Tianjin UniversityTianjin, China; 3 Collaborative Innovation Center of Chemical Science and Engineering (Tianjin)Tianjin, China; 4 School of Environmental and Municipal Engineering, Tianjin Chengjian UniversityTianjin, China; 5 Tianjin Key Laboratory of Aquatic Science and TechnologyTianjin, China

**Keywords:** metabolic engineering, molecular evolution, succinate, synonymous substitution, synthetic biology

## Abstract

Evolution has resulted in thousands of species possessing similar metabolic enzymes with identical functions that are, however, regulated by different mechanisms. It is thus difficult to select optimal gene to engineer novel or manipulated metabolic pathways. Here, we tested the ability of molecular evolutionary analysis to identify appropriate genes from various species. We calculated the fraction of synonymous substitution and the effective number of codons (ENC) for nine genes stemming from glycolysis. Our research indicated that an enzyme gene with a stronger selective constraint in synonymous sites would mainly regulate corresponding reaction flux through altering the concentration of the protein, whereas those with a more relaxed selective constraint would primarily affect corresponding reaction flux by changing kinetic properties of the enzyme. Further, molecular evolutionary analysis was investigated for three types of genes involved in succinate precursor supply by catalysis of pyruvate. In this model, overexpression of *Corynebacterium glutamicum pyc* should result in greater conversion of pyruvate. Succinate yields in two *Escherichia coli* strains that overexpressed each of the three types of genes supported the molecular evolutionary analysis. This approach may thus provide an alternative strategy for selecting genes from different species for metabolic engineering and synthetic biology.

## Introduction

The synonymous codon usage reflects a balance between selective and neutral evolutionary forces. In microorganisms, for a given set of synonymous codons, the relevant tRNAs are not equally abundant (Ikemura 1985). Therefore, there must be a preferred set of codons to match the most abundant tRNAs improving translational efficiency (Kanaya et al. 1999, 2001; Novoa and Ribas de Pouplana 2012). Moreover, synonymous sites may affect the secondary structure of mRNA conferring resistance to premature degradation and therefore selection might against synonymous substitutions that disrupt base pairing (Shen et al. 1999; Duan et al. 2003; Capon et al. 2004; Chamary and Hurst 2005; Novoa and Ribas de Pouplana 2012; Shabalina et al. 2013). Thus, particular codons may be selected to optimize structure or stability. And the stability of mRNA also influences the concentration of protein. The aforementioned factors have presented a level of convergent evidence that, in microorganisms, most synonymous substitution may slightly alter the quantity of proteins by altering the translational process. Recently, Ma et al. (2010) reported that synonymous substitution may be under more relaxed selective pressure when synonymous substitution occur in genes encoding enzymes compared with that occur in genes encoding nonenzyme genes, as regulatory mechanisms may differ between enzyme and nonenzyme genes. The function of enzymes can be regulated by altering their kinetic properties, whereas the function of nonenzyme genes is regulated primarily by altering their expression levels. However, by comparing nearly 70 000 genes, Zhang et al. (X. H. Ma, X. B. Zhang, B. Y. Wang, , Y. F. Mao, Z. W. Wang, T. Chen and X. M. Zhao, personal communication) found that the selective constraint on synonymous sites of some enzyme-encoding genes differs from that of other enzyme-encoding genes, suggesting that some enzymes are primarily regulated by their concentration, whereas other enzymes are regulated by altering their kinetic properties (Wright and Rausher 2010). The different pattern in enzyme regulation may inform the choice of the optimum enzymes for engineering of microorganisms for target chemical production.

In many cases, enhanced production of a target chemical requires the introduction/activation of additional enzymes and/or pathways in the host strain. However, many metabolic genes are highly conserved across many species and there is functional redundancy of metabolic genes within species, it can be difficult to select the most suitable donor species and genes to maximize production. The *in vitro* kinetic properties of enzymes from different species provide some selection criteria (Scheer et al. 2011), but differences between *in vivo* and *in vitro* kinetics are not yet well understood because of the intrinsic complexity of the intracellular environment. Consequently, time-consuming and costly trial-and-error approaches are still widely used (Zheng et al. 2012; Meiswinkel et al. 2013).

Due to increases in global energy consumption and supply concerns, much attention has focused on engineering microorganisms for the production of biofuels, pharmaceuticals, plastics, food products, and more (Balzer et al. 2013). Concerning maximizing yields of bio-based productions by microorganism, as a case study, evolutionary analyses were attempted to assist in engineering *Escherichia coli* for efficient conversion of glucose to succinate. Succinate, a C4-dicarboxylic acid, has a wide range of applications in fields as diverse as agriculture, medicine, polymer synthesis, and chemistry (Ma et al. 2013). *Escherichia coli* is one of the most promising succinate producers because of its well-studied genetics and easy manipulation. Succinate is not the primary product of pyruvate conversion in *E. coli* under aerobic or anaerobic conditions. Thus, it is necessary to redirect metabolic resources to increase succinic acid production and to reduce the formation of other by-products. Toward this goal, a number of metabolic engineering approaches have been developed to increase succinate production in *E. coli* (Jantama et al. 2008a; Jantama et al. 2008b; Cao et al. 2011; Yu et al. 2011; Balzer et al. 2013; Ma et al. 2013; Zhu et al. 2013). These approaches mainly rely either on blocking competitive or succinate degradation pathways (Chatterjee et al. 2001; Zhang et al. 2009; Singh et al. 2010; Balzer et al. 2013), or on activating endogenous or heterologous enzymes (Singh et al. 2010; Ma et al. 2013; Zhu et al. 2013) to direct the carbon flow to oxaloacetate (OAA) or malate, from which succinic acid can be produced.

In *E. coli*, the formation of succinate mainly occurs via the carboxylation of phosphoenolpyruvate (PEP) to form oxaloacetate. Half of the glucose-derived PEP is consumed by the PEP: carbohydrate phosphotransferase system (PTS) to transport glucose across the cell membrane. This metabolic rigidity can be overcome by overexpressing PEP carboxylase (PEPC) and/or PEP carboxykinase (PEPCK) or by inactivating genes of the PTS, but the consequent reduction in glucose absorption results in a slower growth rate and, therefore, less overall succinate productivity or production. Alternatively, succinate production can be significantly enhanced by the overexpression of native *E. coli* malic enzyme or non-native pyruvate carboxylase (encoded by *pyc*), both of which convert pyruvate to 4-carbon succinate precursors (Fig. S1).

However, because both malic enzyme and pyruvate carboxylase genes have been identified in many microorganisms, such as *Lactococcus lactis*, *Rhizobium etli,* and *Bacillus subtilis*, it is difficult to determine the appropriate gene donor species. Overexpression of *pyc* gene from *L. lactis* in SBS110MG, an *E. coli* strain whose *adhE* and *ldhA* genes were removed, increased succinate yield from 0.2 to 1.3 mol/mol (more than six times) (Sánchez et al. 2005). But, overexpression of *pyc* gene from *R. etli* resulted in a 2.7-fold enhancement in succinate production (Gokarn et al. 2001). Overexpression of malic enzyme gene from *E. coli* in NZN110 led its succinate yield increased two to three times (Stols and Donnelly 1997; Hong and Lee 2001). These different results may derive from the differences in genotype of host strains, modification of overexpressed genes, and process of cultivation. What is more possible, however, the difference in the aspects of succinate yield or production stems from the enzyme activity regulation of overexpressed genes from various donor species.

In the present work, we tested the ability of evolutionary genetics information to inform the choice of the optimum enzymes for converting pyruvate to succinate from three species. We analyzed the evolutionary selection on synonymous sites of thirteen genes from three microorganisms, including nine glycolysis genes form *B. subtilis*, *Corynebacterium glutamicum*, and *E. coli*, three types of genes converting pyruvate to OAA or malate, that is, *pyc* from *B. subtilis* and from *C. glutamicum*, the NAD-dependent malic enzyme gene (*maeA)* from *E. coli*, and the NADP-dependent malic enzyme gene (*maeB)* from *E. coli*. Based on our hypothesis, overexpression of *pyc* from *C. glutamicum* would be most effective in converting pyruvate to the succinate precursor 4-carbon metabolites. This prediction was supported by heterologous overexpression of these genes for succinate yield in two *E. coli* strains.

## Materials and methods

### DNA sequences

We investigated the sequences of nine genes that encode glycolysis-related enzymes and three types of genes coding for enzymes involved in the conversion of pyruvate to OAA or malate (Data S1) in *B. subtilis*, *C. glutamicum*, and *E. coli*. We examined these genes in four *B. subtilis* strains, four *C. glutamicum* strains, and five *E. coli* strains (Data S1). DNA sequences were obtained from the National Center for Biotechnology Information (NCBI), the Kyoto Encyclopedia of Genes and Genomes (Kanehisa et al. 2008), and Uniprot (Bairoch et al. 2009). The stop and start codons were conserved in most cases, and therefore, they were not taken into account in this analysis. Sequence accession numbers and codon positions used in the analyses are listed in Data S1. DNA sequences were translated into amino acid sequences and aligned using ClustalW (Larkin et al. 2007) in MEGA 4 (Tamura et al. 2007), followed by manual adjustment.

### Molecular evolutionary analyses

Two methods were used to estimate the selective constraint on synonymous sites of a given gene. The first method was to establish the fraction of synonymous substitution (*Ks*) of each gene using DnaSP v. 4.10 (Rozas et al. 2003). We calculated the *Ks* values for the genes that encode the enzymes involved in glycolysis (Data S1) using gene sequences from an outgroup (Data S1). The significance of differences in *Ks* among genes or among species pairs was assessed by two-way anova of *Ks*.

The second method was to analyze codon bias within genes. We calculated effective number of codon (ENC) values for each gene using DnaSP v. 4.10. The mean ENC values of genes under stronger selective constraint should be lower than those of genes under less selective constraint (Akashi 1994; Stoletzki and Eyre-Walker 2007). Rare-codon analysis was performed by online tool (http://www.genscript.com/cgi-bin/tools/rare_codon_analysis). The significance of differences in ENC among genes or among species pairs was assessed by two-way anova of ENC.

### Strains, media, and growth conditions

*Escherichia coli* strain TOP10 was used for propagation and amplification of plasmids, whereas *E. coli* strain W1485 was used for gene deletion and overexpression assays to assess succinic acid production. The features of the strains, plasmids, and primers used in this study are summarized in Table S1. Wild-type and mutant strains were grown in Luria-Bertani (LB) medium containing 1% (w/v) tryptone, 0.5% (w/v) yeast extract, and 0.5% (w/v) NaCl and supplemented with antibiotics as appropriate for plasmid maintenance or gene deletion (100 μg/mL ampicillin or 20 μg/mL chloramphenicol, respectively).

Cells were aerobically cultured in 500-mL Erlenmeyer flasks containing 100 mL LB medium at 37°C. When the optical density at 600 nm (OD_600_) reached 2.0, cells were harvested by centrifugation at 4000 *g*, washed once with distilled water, and resuspended in 50 mL modified LB medium (supplemented with 10 g/L KHCO_3_, 10 g/L MgCO_3_, and 10 g/L glucose) in a 100-mL bottle sealed with a sterile stopper. Plasmid expression was induced with 2 mm isopropylthio-galactoside (IPTG). Fermentation was performed anaerobically for an additional 12 h at 37°C with the agitation rate set at 200 rpm.

### Plasmid construction and gene inactivation

The *pyc* genes were amplified from *B. subtilis* 168 and *C. glutamicum* ATCC13032 genomic DNA. The *maeA* and *maeB* genes were amplified from *E. coli* W1485 genomic DNA. All genes were inserted into pTrc99z (Zheng et al. 2012), a slightly modified medium-copy plasmid constructed by removing the 14-bp ribosome-binding sites downstream from the *trc* promoter in pTrc99a. The *λ* Red recombination system was used for gene deletions, integration, and removal of antibiotic resistance genes as described (Datsenko and Wanner 2000). Sense primers contain sequences corresponding to the N-terminus of each targeted gene (45 bp) followed by 20 bp corresponding to the FRT-kan-FRT cassette. Antisense primers contain sequences corresponding to the C-terminal region of each targeted gene (45 bp) followed by 20 bp corresponding to the cassette. Amplified DNA fragments were electroporated into *E. coli* strain BW21153 harboring Red recombinase (pKD46). In resulting recombinants (JPJ00), the FRT-kan-FRT cassette replaced the deleted region of the target gene by homologous recombination. Subsequently, an additional PCR was used to amplify upstream-FRT-kan-FRT-downstream cassette (approximately 1000 bp of both sides) of JPJ00 genome. The PCR product was electroporated into *E. coli* strain WD3 harboring Red recombinase (pKD46) to construct JPJ05C strain. Chromosomal deletions and integrations were verified by PCR analyses.

### Analytical techniques

Cell growth was monitored by measuring the OD_600_ with a UV-VIS spectrophotometer (TU-1901, Persee, Beijing, China). To analyze the extracellular metabolites, fermentation products were centrifuged at 13 000 *g* for 1 min, and the supernatant was filtered through a 0.2-μm filter prior to HPLC analysis. The HPLC system (HP1100, Agilent Technologies, Palo Alto, CA, USA) was equipped with an ion exchange Aminex HPX 87-H column (Bio-Rad, Richmond, CA, USA) with 5 mm H_2_SO_4_ as the mobile phase at 0.6 mL/min flow rate, 55°C column temperature, and UV absorption at 210 nm.

## Results

### Synonymous substitution of glycolysis genes

We used two approaches to detect evolutionary selection at synonymous sites, estimating the *Ks* value and an analysis of the ENC. The average *Ks* values for the genes encoding the glycolysis enzymes were listed in Table 1. In *B. subtilis*, the *Ks* values of *pfkA* and *pyk* are higher than other genes. Similarly, *pfkA* and *pyk* in *E. coli* and *C. glutamicum* also have higher *Ks* values than most of their glycolysis-related genes. To statistically assess the variations of *Ks* values among genes and species pairs, we performed two-way anova. As is expected, the results of anova indicate that the differences of the *Ks* among different genes are statistically significant (*F*_*B. subtilis*_ = 211.22, *P* < 0.001, *F*_*C. glutamicum*_ = 1089.51, *P* < 0.001, and *F*_*E. coli*_ = 3716.93, *P* < 0.001, Data S2). The three regulated enzymes of glycolysis are hexokinase, phosphofructokinase (encoded by *pfkA*), and pyruvate kinase (encoded by *pyk*). Their activities are primarily regulated by several metabolites (such as ATP and pyruvate) rather than by altering their concentration. Our results demonstrated that the synonymous substitution rates of genes encoding three regulated enzymes are significantly higher than those of other genes.

**Table 1:** Average *Ks* values for the genes encoding the glycolysis enzymes

	Average *Ks* values		
	
Genes	*Bacillus subtilis*	*Corynebacterium glutamicum*	*Escherichia coli*
Glycolysis genes			
*pgi*	1.23	1.33	0.12
*pfkA*	1.27	1.15	0.51
*fbaA*	0.46	0.43	0.08
*tpiA*	0.35	0.69	0.28
*gapA*	0.19	1.06	0.19
*pgk*	0.33	0.70	0.05
*pgm*	0.59	0.66	0.11
*eno*	0.15	0.53	0.07
*pyk*	1.37	0.91	0.79
Succinic acid converting genes			
*maeA*	–	–	0.30
*maeB*	–	–	1.11
*pyc*	1.85	1.05	–

‘–’, no relevant gene.

The higher *Ks* value of a certain enzyme gene supports the hypothesis that the synonymous sites of this gene are under more relaxed selective constraint. This value also consists with an alternative hypothesis: positive selection or faster evolutionary rate. Therefore, selection on synonymous sites of enzyme-encoding genes can also be examined using ENC analysis to estimate codon usage bias.

### Codon usage of glycolysis genes

Selection on synonymous sites of enzyme-encoding genes can also be examined using ENC analysis to estimate codon usage bias (Akashi 1994; Stoletzki and Eyre-Walker 2007). ENC values range from 20 (maximum bias and selective constraint, i.e., only one synonymous codon is used per amino acid) to 61 (no bias, all synonymous codons are being used equally). The ENC values for the glycolysis enzymes are quite variable (Table 2). With one exception of *pgi*, the average ENC values of the *C. glutamicum pfkA* and *pyk* genes are higher than other glycolysis genes (Table 2). The ENC values of *pfkA* and *pyk* from *B. subtilis* are also higher than most glycolysis-related genes. Similarly, *pfkA* and *pyk* in *E. coli* have higher ENC values than most glycolysis-related genes.

**Table 2:** Effective number of codons (ENC) values for glycolysis genes

	ENC values		
	
Genes	*Bacillus subtilis*	*Corynebacterium glutamicum*	*Escherichia coli*
Glycolysis genes			
*pgi*	45.78	40.24	37.43
*pfkA*	49.41	46.77	34.57
*fbaA*	40.55	29.91	30.26
*tpiA*	41.64	33.82	32.97
*gapA*	34.28	34.91	27.92
*pgk*	41.18	28.71	33.02
*pgm*	47.08	31.73	40.27
*eno*	36.42	28.31	27.40
*pyk*	46.94	38.68	46.35
Succinic acid converting genes			
*maeA*	–	–	43.49
*maeB*	–	–	42.09
*pyc*	52.30	39.36	–

‘–’, no relevant gene.

Using two-way anova, we found that, among the nine glycolysis enzyme genes, there was little variation in codon bias across the four *B. subtilis* strains (*F* = 0. 11, *P* = 0.98, Data S3), implying an evolutionary equilibrium in this regard, or that the rate of evolutionary change in ENC was very slow (Lu and Rausher 2003). The codon biases across glycolysis genes for four *C. glutamicum* strains (*F* = 0.48, *P* = 0.75) and five *E. coli* strain (*F* = 0.16, *P* = 0.96) are also not significant. In contrast, for the three microorganisms, codon usage biases are evident across different genes (*F*_*B. subtilis*_ = 236.60, *P* < 0.001; *F*_*C. glutamicum*_ = 1350.26, *P* < 0.001; *F*_*E. coli*_ = 1956.29, *P* < 0.001; Data S3).

Further, we investigated the correlation between substitution rate and the codon usage of glycolysis genes. Our data show that *Ks* values are strongly positively correlated with the ENC values (for *B. subtilis*, Spearman's rank correlation *r* = 0.84, *P* < 0.001; for *C. glutamicum*, Spearman's rank correlation *r* = 0.82, *P* < 0.001; and for *E. coli*, Spearman's rank correlation *r* = 0.64, *P* < 0.001). The *Ks* values, ENC values, and correlation between *Ks* and ENC suggest that the impact of selective constraints on synonymous sites of enzymes whose activity mainly regulated through altering the concentration of the protein is stronger than that of enzymes whose activity primarily regulated by changing kinetic properties of the enzymes.

We further investigated the fraction of synonymous substitution and the ENC values for three types of genes that catalyze the conversion of pyruvate to malic acid and OAA and eventually to succinate. The average *Ks* values for the genes encoding the four pyruvate-converting enzymes were listed in Table 1. The highest *Ks* was 1.85 (*pyc* of *B. subtilis*), which significantly higher than the lowest *Ks* value (*pyc* of *C. glutamicum*). And the average ENC value of the *C. glutamicum pyc* gene was 39.36, indicating that ∼22 codons were effectively unused, whereas the average ENC value of *B. subtilis pyc* was 52.30, demonstrating that only nine codons were effectively unused (Table 2). The ENC and *Ks* values of *pyc* from *B. subtilis* were all the highest comparing with nine glycolysis-related genes. Similarly, *maeA* and *maeB* in *E. coli* had higher ENC values than all glycolysis-related genes except for *pyk*. In contrast, the ENC and *Ks* values of *pyc* from *C. glutamicum* were lower than that of several other glycolysis-related genes. These results imply that the *pyc* of *C. glutamicum* is under the highest selective constraint, followed by *maeB* of *E. coli*, *maeA* of *E. coli*, and *pyc* from *B. subtilis*.

### Experimental validation of molecular evolutionary analysis results

**Figure 1:**
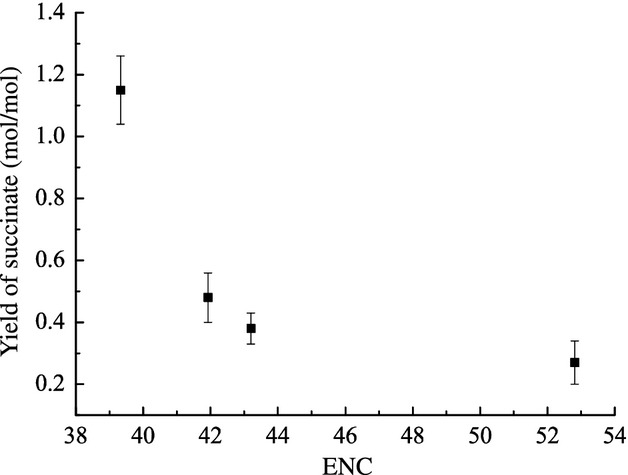
Effects of effective number of codons values on succinate yield of different WD3 strains. The yield of succinate is the molar succinate yield at the end of fermentation (mole of succinate produced per mole of glucose consumed); each value is the mean of three parallel replicates ± standard deviation.

Plasmids carrying the four different genes that encode pyruvate-converting enzymes were introduced into *E. coli* WD3. The ENC values as compared with the yield of succinate from glucose were shown in Fig. 1. ENC values were inversely proportional to succinic acid yield. Notably, the succinate yield from WD3 carrying *pyc* from *C. glutamicum* was approximately three fold higher than that from WD3 carrying *B. subtilis pyc* (*P* < 0.001). The enzyme under higher selective constraint was thus more readily able to convert pyruvate to 4-carbon metabolites.

**Figure 2:**
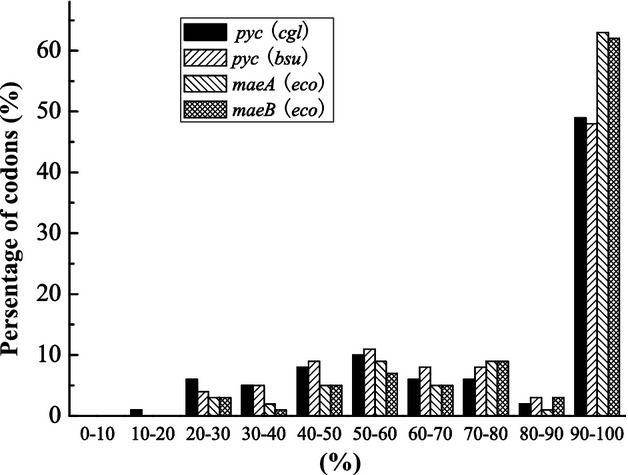
The percentage distribution of codons in computed codon quality groups. The value of 100 is set for the codon with the highest usage frequency for a given amino acid in *Escherichia coli*. Codons with values lower than 30 are likely to hamper the expression efficiency.

The activity of an enzyme can be regulated by changing its abundance and/or by altering its intrinsic catalytic efficiency (Ma et al. 2010; Wright and Rausher 2010). The higher succinate yield in the cells carrying *C. glutamicum pyc* may be due to higher intracellular concentrations of *C. glutamicum* PYC as compared with the other three enzymes. Whereas the four different genes were inserted into identical vectors with the same inducible promoter and translational start sequences, the only factor influencing the intracellular concentration of the four proteins would be their synonymous site codon usage. To further explore how codon usage influences the abundance of these four enzymes, we performed rare-codon analysis. If the higher yield of succinic acid resulted from the presence of fewer rare codons within an overexpressed gene, we would expect to detect fewer rare codons (for *E. coli*) in *pyc* of *C. glutamicum* than in the other three genes. However, our data did not support this hypothesis (Fig. 2). The percentage of rare codons used in the heterologous *pyc* genes from *C. glutamicum* and *B. subtilis* was higher than that in either of the homologous genes *maeA* and *maeB*. Specifically, ∼49.5% of the codons used in the two *pyc* genes were the highest-frequency codon of *E. coli*, whereas ∼19% were lower-frequency codons in *E. coli*. In contrast, 63% of the codons in the *maeA* and *maeB* genes were the highest-frequency codons in *E. coli*, and ∼9.5% were lower-frequency codons in *E. coli*. Polyacrylamide gel electrophoresis of crude protein extracts from the *E. coli* strains further confirmed our deduction. The relative protein expression of *B. subtilis* PYC showed the highest level (2.81), while relative protein expression of *C. glutamicum* PYC was lower, similar with that of *E. coli* MaeA (1.74 vs 1.94). These results suggested that differences in succinate yield are more likely due to differences in the regulation of enzymatic activity.

### Confirmation of the molecular evolutionary analysis results

**Figure 3:**
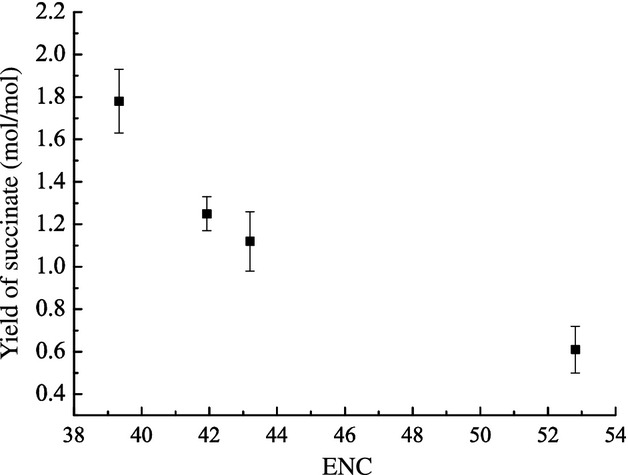
Effects of effective number of codons values on succinate yield of different *pflB*-*focA* deletion strains. The yield of succinate is the molar succinate yield at the end of fermentation (mole of succinate produced per mole of glucose consumed); Each value is the mean of three parallel replicates ± standard deviation.

To examine the effects of overexpression of different genes on glucose-derived succinate yield under different host strain genotype, we deleted the pyruvate formate lyase I–formate channel, a operon (*pflB*-*focA*) from *E. coli* WD3 and individually introduced the four heterologous expressed pyruvate-converting enzymes. Similar tendency was seen in the deletion strains carrying the four heterologous genes: the yield of succinic acid from the four plasmid-carrying *E. coli* strains increased in the order of *B. subtilis pyc* < *E. coli maeA* < *E. coli maeB* < *C. glutamicum pyc* (Fig. 3). Again, higher ENC values of the enzymes corresponded with a lower yield of succinic acid. PflB catalyzes the conversion of pyruvate to acetyl-CoA and formate, and the upstream gene, *focA*, encodes a formate transporter. As predicted, production of formate and acetate was substantially reduced in the deletion strain, and succinate was dramatically increased (Table 3).

**Table 3:** Organic acids profile of the *pflB*-*focA* deletion strains containing different genes

	Yield (mol/mol)*		SUC (g/L)		PYR (g/L)		LAC (g/L)		FOR (g/L)		ACE (g/L)	
						
Plasmids	WD3	JPJ05C	WD3	JPJ05C	WD3	JPJ05C	WD3	JPJ05C	WD3	JPJ05C	WD3	JPJ05C
pTRC99Z + 25	0.27	0.61	1.76	4.02	0.51	0.89	2.41	2.80	1.77	0	3.58	2.03
pTRC99Z + 55	1.15	1.79	7.57	11.73	0.72	0.46	0.22	0.11	1.41	0	0.52	0.12
pTRC99Z + 63	0.48	1.25	3.13	8.19	0.49	0.01	1.81	0.56	1.86	0	3.42	0.88
pTRC99Z + 68	0.38	1.12	2.50	7.34	0.51	0.03	2.75	2.32	1.28	0	3.03	1.49

SUC, succinate; PYR, pyruvate; LAC, lactate; FOR, formate; ACE, acetate.

* Yield of succinate from glucose.

## Discussion

Large-scale genome sequencing of microorganisms now allows researchers to apply sequence-based evolutionary analysis approaches to microbial ecology questions. To our knowledge, this is the report of the use of evolutionary analyses to assist in the engineering of microorganisms for chemical production.

Our *Ks* and ENC analyses suggested that synonymous sites within *pfkA* and *pyk* genes from three microorganisms are under more relaxed selective constraint than those within genes for the other glycolysis enzymes that we examined. A possible explanation for this difference in selective constraint is that *pfkA* and *pyk* are primarily regulated by altering their kinetic properties, whereas the activities of other glycolysis genes are mainly regulated at the level of protein concentration. Further investigation of four enzymes that catalyze the conversion of pyruvate to malic acid and OAA and eventually to succinate demonstrate that *pyc* of *C. glutamicum* is under the highest selective constraint, followed by *maeB* of *E. coli*, *maeA* of *E. coli*, and *pyc* from *B. subtilis*. This result implies that *C. glutamicum* PYC is primarily regulated at the level of protein concentration, whereas the activity of *B. subtilis* PYC is mainly regulated by altering its kinetic properties.

In the Michaelis-Menten model, if the function of an enzyme is primarily regulated by altering its kinetic properties, it will have a wider *K*_*m*_ range, and thus, synonymous substitution will have little impact on the flux of the reaction. In contrast, if the function of an enzyme is primarily regulated by its concentration, it will have a narrower *K*_*m*_ range, and synonymous substitutions that affect intracellular concentration of the enzyme will alter the flux of the reaction. Our rare-codon analysis, SDS-PAGE experiments, and results with *E. coli* strains that overexpressed *pyc* from *C. glutamicum* and *B. subtilis* support the hypothesis that the activities of these two enzymes are regulated by different mechanisms. The activity of *pyc* from *C. glutamicum* appears to be primarily regulated by changing the concentration of the enzyme, whereas the activity of *pyc* from *B. subtilis* appears to be regulated by altering its kinetic properties. In this study, the two *pyc* genes and two malic enzyme genes that we overexpressed in *E. coli* were under the same heterologous transcriptional and translational control. Therefore, differences in succinate production from the four overexpressing strains are likely to be due to the different kinetic properties of these enzymes.

## Data archiving statement

Data for this study are available as Supplementary Material online.

## Supporting Information

**Data S1.** Entry number for analyzed genes from different strains.

**Data S2.** anova analysis of *Ks* values for glycolysis genes.

**Data S3.** anova analysis of ENC values for glycolysis.

**Figure S1.** Metabolic network of succinate producing *Escherichia coli*.

**Table S1.** Strains, plasmids and primers.

## References

[b1] Akashi H (1994). Synonymous codon usage in *Drosophila melanogaster*: natural selection and translational accuracy. Genetics.

[b2] Bairoch A, Consortium U, Bougueleret L, Altairac S, Amendolia V, Auchincloss A, Argoud-Puy G (2009). The universal protein resource (UniProt) 2009. Nucleic Acids Research.

[b3] Balzer GJ, Thakker C, Bennett GN, San KY (2013). Metabolic engineering of *Escherichia coli* to minimize byproduct formate and improving succinate productivity through increasing NADH availability by heterologous expression of NAD^+^-dependent formate dehydrogenase. Metabolic Engineering.

[b4] Cao Y, Cao Y, Lin X (2011). Metabolically engineered *Escherichia coli* for biotechnological production of four-carbon 1,4-dicarboxylic acids. Journal of Industrial Microbiology & Biotechnology.

[b5] Capon F, Allen MH, Ameen M, Burden AD, Tillman D, Barker JN, Trembath RC (2004). A synonymous SNP of the corneodesmosin gene leads to increased mRNA stability and demonstrates association with psoriasis across diverse ethnic groups. Human Molecular Genetics.

[b6] Chamary JV, Hurst L (2005). Evidence for selection on synonymous mutations affecting stability of mRNA secondary structure in mammals. Genome Biology.

[b7] Chatterjee R, Millard CS, Champion K, Clark DP, Donnelly MI (2001). Mutation of the *ptsG* gene results in increased production of succinate in fermentation of glucose by *Escherichia coli*. Applied and Environmental Microbiology.

[b8] Datsenko KA, Wanner BL (2000). One-step inactivation of chromosomal genes in *Escherichia coli* K-12 using PCR products. Proceedings of the National Academy of Sciences of the United States of America.

[b9] Duan J, Wainwright MS, Comeron JM, Saitou N, Sanders AR, Gelernter J, Gejman PV (2003). Synonymous mutations in the human dopamine receptor D2 (DRD2) affect mRNA stability and synthesis of the receptor. Human Molecular Genetics.

[b10] Gokarn RR, Evans JD, Walker JR, Martin SA, Eiteman MA, Altman E (2001). The physiological effects and metabolic alterations caused by the expression of *Rhizobium etli* pyruvate carboxylase in *Escherichia coli*. Applied Microbiology and Biotechnology.

[b11] Hong SH, Lee SY (2001). Metabolic flux analysis for succinic acid production by recombinant *Escherichia coli* with amplified malic enzyme activity. Biotechnology and Bioengineering.

[b12] Ikemura T (1985). Codon usage and tRNA content in unicellular and multicellular organisms. Molecular Biology and Evolution.

[b13] Jantama K, Haupt MJ, Svoronos SA, Zhang X, Moore JC, Shanmugam KT, Ingram LO (2008a). Combining metabolic engineering and metabolic evolution to develop nonrecombinant strains of *Escherichia coli* C that produce succinate and malate. Biotechnology and Bioengineering.

[b14] Jantama K, Zhang X, Moore JC, Shanmugam KT, Svoronos SA, Ingram LO (2008b). Eliminating side products and increasing succinate yields in engineered strains of *Escherichia coli* C. Biotechnology and Bioengineering.

[b15] Kanaya S, Yamada Y, Kudo Y, Ikemura T (1999). Studies of codon usage and tRNA genes of 18 unicellular organisms and quantification of *Bacillus subtilis* tRNAs: gene expression level and species-specific diversity of codon usage based on multivariate analysis. Gene.

[b16] Kanaya S, Kinouchi M, Abe T, Kudo Y, Yamada Y, Nishi T, Mori H (2001). Analysis of codon usage diversity of bacterial genes with a self-organizing map (SOM): characterization of horizontally transferred genes with emphasis on the *E. coli* O157 genome. Gene.

[b17] Kanehisa M, Araki M, Goto S, Hattori M, Hirakawa M, Itoh M, Katayama T (2008). KEGG for linking genomes to life and the environment. Nucleic Acids Research.

[b18] Larkin MA, Blackshields G, Brown NP, Chenna R, McGettigan PA, McWilliam H, Valentin F (2007). Clustal W and clustal X version 2.0. Bioinformatics.

[b19] Lu YQ, Rausher MD (2003). Evolutionary rate variation in anthocyanin pathway genes. Molecular Biology and Evolution.

[b20] Ma X, Wang Z, Zhang X (2010). Evolution of dopamine-related systems: biosynthesis, degradation and receptors. Journal of Molecular Evolution.

[b21] Ma J, Gou D, Liang L, Liu R, Chen X, Zhang C, Zhang J (2013). Enhancement of succinate production by metabolically engineered *Escherichia coli* with co-expression of nicotinic acid phosphoribosyltransferase and pyruvate carboxylase. Applied Microbiology and Biotechnology.

[b22] Meiswinkel TM, Gopinath V, Lindner SN, Nampoothiri KM, Wendisch VF (2013). Accelerated pentose utilization by *Corynebacterium glutamicum* for accelerated production of lysine, glutamate, ornithine and putrescine. Microbial biotechnology.

[b23] Novoa EM, Ribas de Pouplana L (2012). Speeding with control: codon usage, tRNAs, and ribosomes. Trends in Genetics.

[b24] Rozas J, Sanchez-DelBarrio JC, Messeguer X, Rozas R (2003). DnaSP, DNA polymorphism analyses by the coalescent and other methods. Bioinformatics.

[b25] Sánchez AM, Bennett GN, San K-Y (2005). Efficient succinic acid production from glucose through overexpression of pyruvate carboxylase in an *Escherichia coli* alcohol dehydrogenase and lactate dehydrogenase mutant. Biotechnology progress.

[b26] Scheer M, Grote A, Chang A, Schomburg I, Munaretto C, Rother M, Söhngen C (2011). BRENDA, the enzyme information system in 2011. Nucleic Acids Research.

[b27] Shabalina SA, Spiridonov NA, Kashina A (2013). Sounds of silence: synonymous nucleotides as a key to biological regulation and complexity. Nucleic Acids Research.

[b28] Shen LX, Basilion JP, Stanton VP (1999). Single-nucleotide polymorphisms can cause different structural folds of mRNA. Proceedings of the National Academy of Sciences of the United States of America.

[b29] Singh A, Soh Cher K, Hatzimanikatis V, Gill RT (2010). Manipulating redox and ATP balancing for improved production of succinate in *E. coli*. Metabolic Engineering.

[b30] Stoletzki N, Eyre-Walker A (2007). Synonymous codon usage in *Escherichia coli*: selection for translational accuracy. Molecular Biology and Evolution.

[b31] Stols L, Donnelly M (1997). Production of succinic acid through overexpression of NAD ^(+)^-dependent malic enzyme in an *Escherichia coli* mutant. Applied and Environmental Microbiology.

[b32] Tamura K, Dudley J, Nei M, Kumar S (2007). MEGA4: molecular evolutionary genetics analysis (MEGA) software version 4.0. Molecular Biology and Evolution.

[b33] Wright KM, Rausher MD (2010). The evolution of control and distribution of adaptive mutations in a metabolic pathway. Genetics.

[b34] Yu C, Cao Y, Zou H, Xian M (2011). Metabolic engineering of *Escherichia coli* for biotechnological production of high-value organic acids and alcohols. Applied Microbiology and Biotechnology.

[b35] Zhang X, Jantama K, Shanmugam KT, Ingram LO (2009). Reengineering *Escherichia coli* for succinate production in mineral salts medium. Applied and Environmental Microbiology.

[b36] Zheng Z, Chen T, Zhao M, Wang Z, Zhao X (2012). Engineering *Escherichia coli* for succinate production from hemicellulose via consolidated bioprocessing. Microbial Cell Factories.

[b37] Zhu LW, Li XH, Zhang L, Li HM, Liu JH, Yuan ZP, Chen T (2013). Activation of glyoxylate pathway without the activation of its related gene in succinate-producing engineered *Escherichia coli*. Metabolic Engineering.

