# Supplementary material for: Engineering microorganisms based on molecular evolutionary analysis: a succinate production case study
Source: Evol Appl. 2014 Sep 2;7(8):913–20. doi: 10.1111/eva.12186 (PMC4211721; doi:10.1111/eva.12186)
Supplement: Supplementary file 4 — Figure S1. Metabolic network of succinate producing Escherichia coli. [file eva0007-0913-sd4.docx]

**Supplementary Figure**


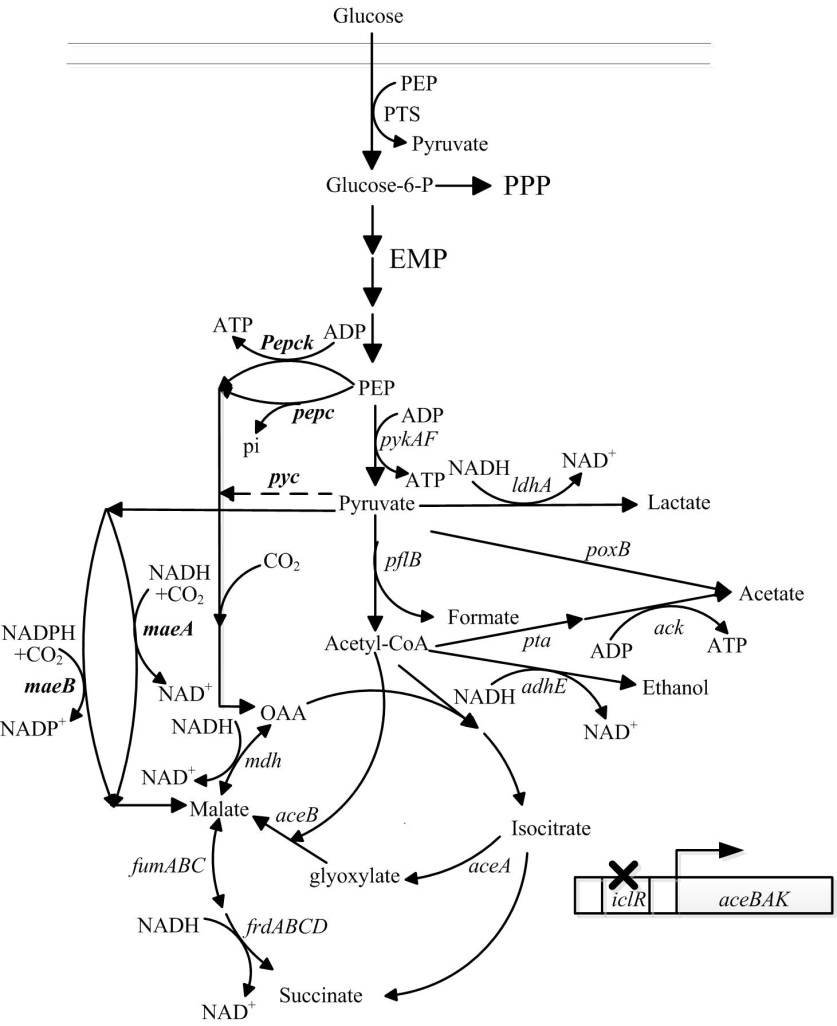


Figure S1 Metabolic network of succinate producing *E. coli*

PEP, phosphoenolpyruvate; PTS, PEP: carbohydrate phosphotransferase system; PPP, Pentose Phosphate Pathway; EMP, Embden-Meyerhof-Parnas pathway; solid arrow means native gene or reaction in *E. coli* and dotted arrow means introduced heterologous one
