# Supplementary material for: Engineering microorganisms based on molecular evolutionary analysis: a succinate production case study
Source: Evol Appl. 2014 Sep 2;7(8):913–20. doi: 10.1111/eva.12186 (PMC4211721; doi:10.1111/eva.12186)
Supplement: Supplementary file 5 — Table S1. Strains, plasmids and primers. [file eva0007-0913-sd5.docx]

**Supplementary Table**

**Table S1:** Strains, plasmids and primers

|  | Description | Origin |
| --- | --- | --- |
| **Strains** |  |  |
| W1485 | Wild type, donor of *maeA* and *maeB* genes | Laboratory Stock |
| BW21153 | Wild type | Laboratory Stock |
| WD3 | W1485 Δ*ldhA::*FRT Δ*adhE::*FRT Δ*ackA::*FRT | Laboratory Stock |
| JPJ00C | BW21153Δ*pfl-focA::*FRT*::cat::*FRT | This work |
| JPJ05C | W1485 Δ*ldhA::*FRT Δ*adhE::*FRT Δ*ackA::*FRT Δ*pfl-focA::*FRT*::*Cat*::*FRT | This work |
| TOP10 | Host for plasmid construction | Laboratory Stock |
| *Bacillus subtilis* 168 | Wild type, *pyc* gene donor | Laboratory Stock |
| *Corynebacterium glutamicum*ATCC13032 | Wild type, *pyc* gene donor | Laboratory Stock |
|  |  |  |
| **Plasmids** |  |  |
| pUC18 | Amp^r^ | Laboratory Stock |
| pKD46 | Amp^r^,*γ β exo* (Red recombinase), temperature-conditional replicon | Laboratory Stock |
| pCP20 | Amp^r^, Cm^r^,*ts-rep*, [FLP], [CI857](*lamda*)(*ts*) | Laboratory Stock |
| pKD4 | Amp^r^,FRT-*kan*-FRT | Laboratory Stock |
| pKD3 | Amp^r^,FRT-*kan*-FRT | Laboratory Stock |
| pTRC99z | Amp^r^, middle copy expression plasmid with *trc* promoter | Laboratory Stock |
| pTRC99z +25 | Amp^r^, containg *pyc*from *B. subtilis* 168 | This work |
| pTRC99z +68 | Amp^r^, containg *maeA*from *E. coli* W1485 | This work |
| pTRC99z +63 | Amp^r^, containg *maeB* from *E. coli* W1485 | This work |
| pTRC99z +55 | Amp^r^, containg *pyc*from *C. glutamicum* ATCC13032 | This work |
|  |  | |
| **Primers** | Sequence (5'-3') | |
| MXH25-up | GCACGGGTACCAGGAGGAGGTATGTCTCAGCAATCGATACAAAAAG | |
| MXH25-down | GGGCGGTCGACTTATCATCACGCTTTTTCAATTTCAAGGAGCAGAT | |
| MXH55-up | CTTGCGAGCTCTGACAGTAGGAGGAGGTCTAATGTCGACTCACACATCTTCAACG | |
| MXH55-down | GCACGGATCCATGTTAGGCGGTTTAGGAAACGACGACGATC | |
| MXH63-1 | TAGATGAGCTCAGGAGGAACAACCAAATGGATGACCAGTT | |
| MXH63-2 | TAGATGGATCCTATTCAGGGTAAGCGTGAGAGTT | |
| MXH68-1 | CTCAGGAGCTCGAGTGAGTGACATGGAACCAAA | |
| MXH68-2 | CTCAGGGTACCGAAATAGCCCGGTAGCCTTCAC | |
| MXH221-UP | ATAGATTGAGTGAAGGTACGAGTAATAACGTCCTGCTGCTGTTCTTGTGTAGGCTGGAGCTGCTT | |
| MXH221-DOWN | GCTTTGTTAGTATCTCGTCGCCGACTTAATAAAGAGAGAGTTAGTATGGGAATTAGCCATGGTCC | |
| MXH52-1 | GTATTATCGTGCCTGGCATGTCCGA | |
| MXH52-4 | GCCTTTCACGCGTTCCATGGTCTCT | |

Note: MXH25-UP and MXH25-down were used to amplify the *pyc* gene from *B. subtilis*, MXH55-UP and MXH55-down were used to amplify the *pyc* gene of *C. glutamicum*, MXH63-1 and MXH63-2 were used to amplify the *maeB* gene of *E.coli*, and MXH68-1 and MXH68-2 were used to amplify the *maeA* gene from *E.coli*.

MXH221-up and MXH221-down were used to remove *pfl-focA* gene from BW21153. MXH52-1 and MXH52-4 were used to amplify upstream-FRT-kan-FRT-downstream cassette (approximately 1000bp of both sides) of JPJ00 genome (See Materials and Methods).
